# Turkish Cross-Cultural Adaptation of the BRIEF-MPI in Older Adults with Cancer: Convergent and Discriminative Validity Compared with the G8 and Clinical Frailty Scale

**DOI:** 10.3390/healthcare14172813

**Published:** 2026-09-02

**Authors:** Sercan On, Haydar Cagatay Yuksel, Semih Sergen Semerci, Ecem Ozdemir, Mustafa Kagan Ozdel, Fatma Ozge Kayhan Kocak

**Affiliations:** 1 Department of Medical Oncology, Ege University Faculty of Medicine, Izmir 35100, Turkey; 2 Department of Internal Medicine, Ege University Faculty of Medicine, Izmir 35100, Turkey; 3 Medical Intern, Ege University Faculty of Medicine, Izmir 35100, Turkey; 4 Division of Geriatrics, Department of Internal Medicine, Izmir Tepecik Education and Research Hospital, Health Science University, Izmir 35020, Turkey; drozgekayhankocak@gmail.com

**Keywords:** geriatric oncology, frailty, comprehensive geriatric assessment, BRIEF-MPI, G8, older adults, cancer

## Abstract

**Background:** Older adults with cancer represent a heterogeneous population with varying levels of frailty and multidimensional vulnerability. Rapid screening tools are needed to identify patients who may benefit from comprehensive geriatric assessment (CGA). This study aimed to translate and culturally adapt the Brief Multidimensional Prognostic Index (BRIEF-MPI) into Turkish and evaluate its performance in older adults with cancer. **Methods:** This cross-sectional methodological study included 177 patients aged ≥65 years with solid malignancies. The BRIEF-MPI was translated and culturally adapted using a structured cross-cultural adaptation process. Clinical performance was evaluated by comparison with the G8 screening tool and the Clinical Frailty Scale (CFS). Correlations with frailty-related measures were assessed using Spearman’s rank correlation. Receiver operating characteristic (ROC) analyses were performed to evaluate discriminative performance for identifying CFS-defined frailty. **Results:** The mean age was 72.5 ± 5.8 years, and 56.5% of participants were women. A positive BRIEF-MPI screening (classes 2–3) was identified in 17.5% of participants, whereas 50.8% screened positive according to the G8. Both instruments were significantly associated with CFS-defined frailty (p<0.001). BRIEF-MPI demonstrated an AUC of 0.883 (95% CI: 0.797–0.969) and higher specificity (91.5%) than the G8 (56.9%), while the G8 showed higher sensitivity (100%). BRIEF-MPI scores were significantly associated with distant metastasis, palliative treatment intent, and poorer ECOG performance status (all p≤0.001). **Conclusions:** The Turkish version of the BRIEF-MPI demonstrated good clinical performance for identifying frailty and multidimensional vulnerability in older adults with cancer. Although its overall discriminative ability was comparable to that of the G8, its higher specificity suggests potential utility in clinical settings where reducing false-positive referrals is prioritized. Future prospective and implementation studies are needed to evaluate its clinical utility, prognostic value, and actual impact on geriatric referral pathways in routine oncology practice.

## 1. Introduction

Older adults account for a rapidly growing proportion of the global cancer population, yet substantial heterogeneity exists in physiological reserve, functional capacity, comorbidity burden, cognition, nutrition, and social support among individuals of the same chronological age. Consequently, treatment decisions in geriatric oncology increasingly rely on individualized, multidimensional assessments rather than on chronological age alone. Current oncology guidelines, therefore, recommend the use of geriatric screening tools to identify vulnerable older adults who may benefit from comprehensive geriatric assessment (CGA) before treatment initiation [1,2].

Despite the recognized benefits of CGA, including improved treatment tolerance, reduced toxicity, and optimization of supportive care interventions, the routine implementation of a full geriatric assessment remains challenging in daily oncology practice due to time constraints, workforce limitations, and resource requirements. As a result, brief geriatric screening instruments have become increasingly important for the initial identification of frailty and multidimensional vulnerability in older adults with cancer [3,4].

The Brief Multidimensional Prognostic Index (BRIEF-MPI) is a rapid multidimensional screening instrument derived from the Comprehensive Geriatric Assessment. It evaluates multiple geriatric domains, including functional status, cognition, mobility, nutrition, comorbidity burden, medication use, and social status within approximately five minutes. Previous studies have demonstrated good agreement between the BRIEF-MPI and the full Multidimensional Prognostic Index, as well as associations with adverse clinical outcomes across different clinical settings. Furthermore, emerging evidence suggests that the BRIEF-MPI may provide multidimensional vulnerability stratification comparable to that of commonly used frailty screening tools while preserving clinically relevant geriatric domains [5,6,7]. Beyond prognostic stratification, emerging evidence suggests that the BRIEF-MPI may also help identify multidimensional geriatric vulnerabilities requiring personalized preventive and supportive interventions in older adults [8,9].

However, evidence regarding the clinical performance of the BRIEF-MPI in geriatric oncology populations remains limited, and the instrument has not previously undergone cross-cultural adaptation or clinical evaluation in Turkish older adults with cancer. In particular, its ability to identify frailty and multidimensional vulnerability, compared with commonly used screening approaches, has not been established.

Therefore, the aim of the present study was to translate and culturally adapt the BRIEF-MPI into Turkish and to evaluate its clinical performance in older adults with cancer by comparing it with established geriatric screening and frailty measures, including the G8 screening tool and the Clinical Frailty Scale.

## 2. Methods

This cross-sectional methodological study included adults aged 65 years and older with a confirmed diagnosis of solid malignancy who attended the Medical Oncology Outpatient Clinic at Ege University Faculty of Medicine between 10 March 2026 and 8 May 2026. Patients who were able to communicate and provide informed consent were consecutively recruited.

All eligible patients presenting during the predefined study period were consecutively recruited. A post hoc sample-size adequacy calculation for the ROC analysis was performed using MedCalc software version 22.016 (MedCalc Software Ltd., Ostend, Belgium) (based on the Hanley and McNeil method). Assuming an expected area under the curve (AUC) of 0.72 based on prior literature comparing the BRIEF-MPI with the Clinical Frailty Scale (CFS), an alpha of 0.05, 80% statistical power, and the observed negative-to-positive ratio of 6.37 (153 non-frail to 24 frail participants), a minimum sample size of 111 participants was required [7]. Thus, our final enrolled cohort of 177 consecutive patients provided adequate statistical power for the discriminative analyses.

### 2.1. Data Collection

Sociodemographic and clinical characteristics were recorded, including age, sex, educational status, marital status, and living arrangements.

Frailty and geriatric vulnerability were assessed using the Turkish version of the BRIEF Multidimensional Prognostic Index (BRIEF-MPI), the G8 screening tool, and the Clinical Frailty Scale (CFS).

The CFS is a validated clinical instrument used to assess frailty-related vulnerability and functional decline. The scale ranges from 1 (very fit) to 9 (terminally ill), based on pictorial representations and descriptions of functional performance and dependency. Higher scores indicate greater frailty. The Turkish validity of the CFS has previously been established, and the instrument is widely used in geriatric and oncology settings [10,11].

The G8 Screening Tool is a validated geriatric oncology screening tool developed to identify older adults with cancer who may benefit from a comprehensive geriatric assessment. The instrument consists of eight items evaluating nutritional status, weight loss, mobility, neuropsychological problems, body mass index, polypharmacy, self-perceived health, and age. Total scores range from 0 to 17, with scores ≤ 14 indicating increased vulnerability and the need for comprehensive geriatric assessment [12].

The Brief Multidimensional Prognostic Index (BRIEF-MPI) is a rapid multidimensional screening instrument derived from the Comprehensive Geriatric Assessment (CGA), designed to evaluate multidimensional geriatric vulnerability in approximately five minutes. The instrument assesses eight geriatric domains: basic activities of daily living (ADL), instrumental activities of daily living (IADL), mobility, cognitive status, nutritional status, comorbidity burden, number of medications, and cohabitation status. Each domain is categorized into low (0), moderate (0.5), or high risk (1), and the final BRIEF-MPI score is calculated by summing the domain scores and dividing the total by eight, yielding a continuous score ranging from 0.00 to 1.00, with higher scores indicating greater multidimensional vulnerability and poorer prognosis. Based on validated thresholds, participants are categorized into three risk groups: low risk (0.00–0.33), moderate risk (0.34–0.66), and high risk (≥0.67). For classification analyses, participants in the moderate- and high-risk categories (BRIEF-MPI classes 2–3) were classified as BRIEF-MPI positive, while those in the low-risk category (class 1) were classified as BRIEF-MPI negative. Previous studies have demonstrated strong agreement between the BRIEF-MPI and the full Multidimensional Prognostic Index, as well as its prognostic and discriminative validity across different clinical settings [5].

### 2.2. Turkish Translation and Cross-Cultural Adaptation of the BRIEF-MPI

The Turkish adaptation of the BRIEF-MPI was conducted using a source-instrument-based approach to preserve semantic and conceptual equivalence with the original instrument. Permission to use and adapt the BRIEF-MPI was obtained from the original developers before initiation of the study.

As the BRIEF-MPI is a composite multidimensional instrument comprising selected items derived from established geriatric assessment measures, the source of each domain and item was first identified from the original BRIEF-MPI framework. For domains derived from established assessment instruments, previously validated Turkish versions of the corresponding source instruments were used to guide the adaptation whenever available. Specifically, validated Turkish versions of the Katz Activities of Daily Living, Lawton–Brody Instrumental Activities of Daily Living, Short Portable Mental Status Questionnaire, Mini Nutritional Assessment, and Modified Barthel Index were used for the corresponding functional, cognitive, nutritional, and mobility items. The wording of these items was harmonized with the item structure, response options, and scoring system of the original BRIEF-MPI rather than independently re-translating the source items from English. For the mobility domain, the original BRIEF-MPI uses three items based on the Barthel mobility scale—transfer, walking, and stair climbing—with item selection informed by the MPI-InChianti approach. The comorbidity, medication, and cohabitation domains were adapted in accordance with the structure and scoring framework of the original BRIEF-MPI.

The resulting preliminary Turkish BRIEF-MPI was subsequently reviewed by an expert panel comprising one oncologist, one geriatrician, and one internal medicine specialist to assess semantic, conceptual, and cultural appropriateness. No quantitative content-validity index was calculated.

To evaluate clarity and comprehensibility, the draft version was pilot tested with 15 consecutively recruited older adults from diverse educational and socioeconomic backgrounds. Participant feedback regarding item comprehension was obtained during pilot administration; formal cognitive interviewing or structured debriefing was not performed. No substantive linguistic, conceptual, cultural, or scoring modifications were required following pilot testing.

A detailed item-level mapping of the original BRIEF-MPI items, their source instruments, the validated Turkish sources where applicable, and the final Turkish wording is provided in Supplementary File S1. The finalized Turkish BRIEF-MPI form is provided in Supplementary File S2.

### 2.3. Statistical Analysis

Statistical analyses were performed using SPSS version 25 (IBM Corp., Armonk, NY, USA). The distribution of continuous variables was assessed using the Kolmogorov–Smirnov test. Continuous variables were expressed as mean ± standard deviation (SD) or median (interquartile range [IQR]), as appropriate, while categorical variables were presented as frequencies and percentages.

Clinical performance and convergent validity of the BRIEF-MPI were evaluated through comparison with established frailty and geriatric screening measures. Convergent validity was assessed by examining associations between BRIEF-MPI total scores, G8 scores, and frailty-related measures, including the Clinical Frailty Scale (CFS), Katz Activities of Daily Living (ADL), Lawton–Brody Instrumental Activities of Daily Living (IADL), and age using Spearman’s rank correlation coefficient.

Exploratory unadjusted comparisons of BRIEF-MPI total scores according to clinically relevant oncological characteristics, including distant metastasis status, treatment intent, and Eastern Cooperative Oncology Group (ECOG) performance status, were performed using the Mann–Whitney U test. No multivariable adjustment was performed for these analyses.

To evaluate the association of the predefined BRIEF-MPI and G8 classifications with CFS-defined frailty, BRIEF-MPI classes 2–3 were classified as positive, and G8 vulnerability was defined as a G8 score ≤ 14 points. Frailty status, defined as CFS ≥ 5, was used as the reference classification. Associations between categorical frailty classifications were examined using the chi-square test, and effect sizes were quantified using Cramer’s V.

Receiver operating characteristic (ROC) curve analyses were performed to evaluate the discriminative performance of BRIEF-MPI and G8 for identifying CFS-defined frailty. Because higher BRIEF-MPI scores indicate greater vulnerability, whereas lower G8 scores indicate greater vulnerability, the G8 score was reversed for ROC analysis so that higher values indicated greater vulnerability for both instruments. Diagnostic performance was assessed using the area under the curve (AUC) with corresponding 95% confidence intervals (95% CI). Sensitivity, specificity, positive and negative predictive values, and positive and negative likelihood ratios were calculated with corresponding 95% confidence intervals using the predefined clinical classifications. To formally compare the discriminative performance of BRIEF-MPI and G8, differences between correlated ROC curves were assessed using the DeLong test.

Accordingly, the present analyses were intended to provide evidence of convergent and discriminative validity rather than comprehensive psychometric or prognostic validation.

Because the BRIEF-MPI is a multidimensional composite clinical index comprising conceptually heterogeneous domains rather than a psychometric instrument measuring a single latent construct, internal consistency measures (e.g., Cronbach’s alpha) and factor analysis were not considered methodologically appropriate and were therefore not performed, consistent with the methodology of the original BRIEF-MPI validation studies. A two-sided *p*-value < 0.05 was considered statistically significant.

## 3. Results

### 3.1. Participant Characteristics

A total of 177 older adults with cancer were included in the study. The mean age was 72.5 ± 5.8 years, and 56.5% were women. Most participants were married (70.1%), had 5–8 years of education (49.7%), and were living with a spouse/partner (67.2%).

Regarding oncological characteristics, the most common cancer types were breast cancer (29.4%), colorectal cancer (13.0%), and prostate cancer (11.9%). Distant organ metastasis was present in 40.1% of participants. Approximately half of the cohort was receiving active systemic antineoplastic treatment (42.4%) or was under follow-up/surveillance (48.6%), while 59.3% were being treated with curative intent and 40.7% with palliative intent. Most participants (65.0%) had been diagnosed with cancer for more than 12 months.

Functional and geriatric assessment findings showed mean Katz ADL and Lawton–Brody IADL scores of 5.4 ± 1.2 and 6.9 ± 2.0, respectively. The mean Clinical Frailty Scale (CFS) score was 3.2 ± 1.4, while the mean G8 score was 13.7 ± 2.5 and the mean BRIEF-MPI total score was 0.22 ± 0.15. According to predefined BRIEF-MPI categories, 82.5% of participants were classified as low risk, 16.9% as moderate risk, and 0.6% as high risk. Overall, 17.5% of participants were classified as BRIEF-MPI positive (classes 2–3), compared with 50.8% who screened positive for vulnerability according to the G8 (≤14 points) (Table 1).

### 3.2. Frailty Identification and Discriminative Performance

Both BRIEF-MPI moderate/high-risk classification (classes 2–3) and G8 vulnerability (≤14 points) showed significant associations with CFS-defined frailty (both *p* < 0.001) (Table 2). However, the magnitude of association was stronger for BRIEF-MPI classification than for G8 classification, with a Cramer’s V of 0.599 versus 0.389, respectively.

**Figure 1:**
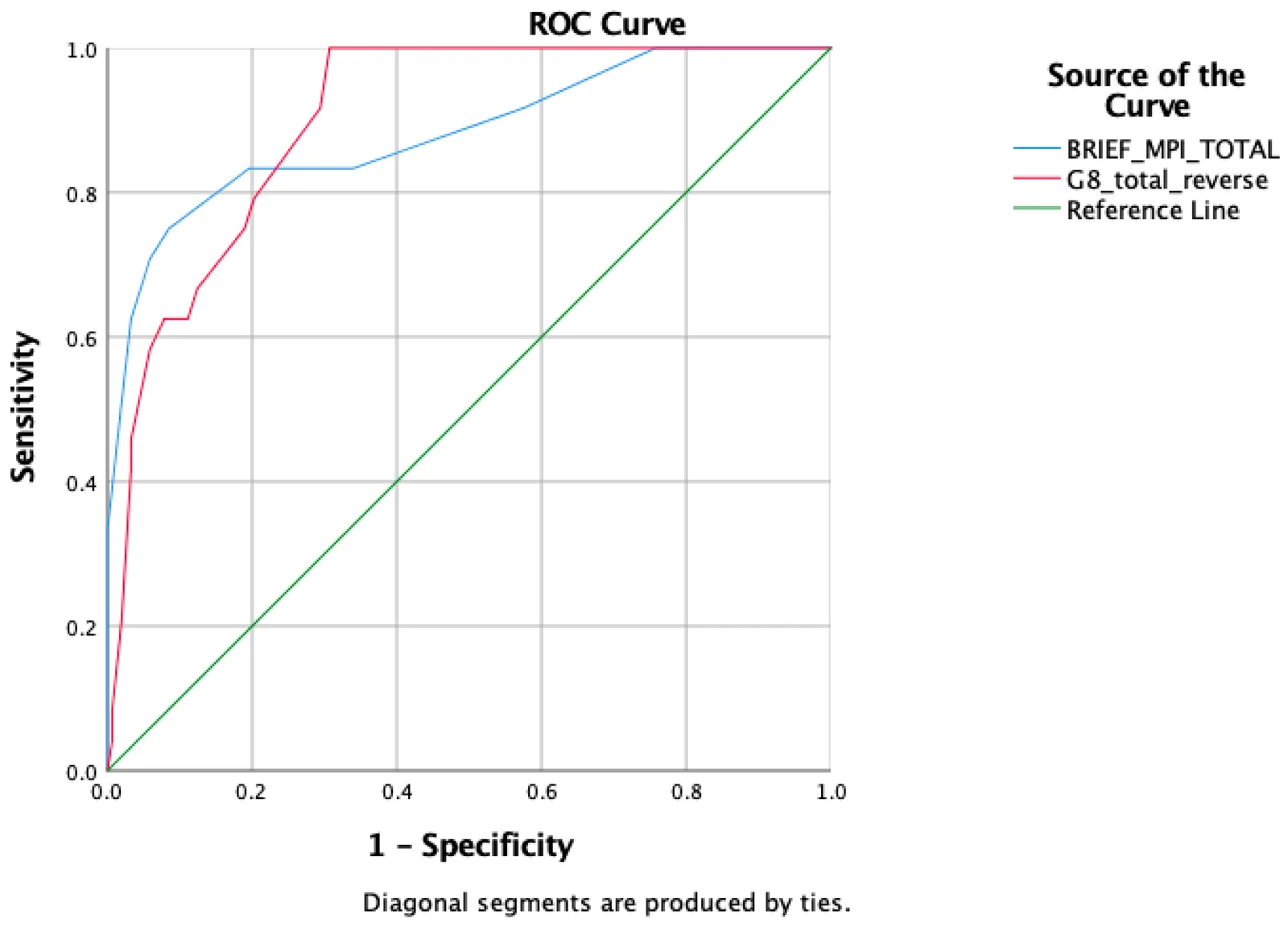
Discriminative performance of continuous BRIEF-MPI and G8 total scores for identifying Clinical Frailty Scale-defined frailty (CFS ≥ 5). Receiver operating characteristic (ROC) curve analysis demonstrated an AUC of 0.883 (95% CI: 0.797–0.969) for the BRIEF-MPI and 0.900 (95% CI: 0.849–0.951) for the G8.

ROC analyses using the continuous total scores demonstrated good discrimination for identifying CFS-defined frailty (CFS ≥ 5). The BRIEF-MPI total score yielded an AUC of 0.883 (95% CI: 0.797–0.969), while the G8 total score yielded an AUC of 0.900 (95% CI: 0.849–0.951) (Figure 1). Comparison of the two correlated ROC curves using the DeLong test showed no statistically significant difference between the AUCs (*p* = 0.637).

At the predefined clinical thresholds, the BRIEF-MPI moderate/high-risk classification showed a sensitivity of 75.0% (95% CI: 53.3–90.2) and a specificity of 91.5% (95% CI: 85.9–95.4) for CFS-defined frailty. In comparison, the G8 threshold of ≤14 points showed a sensitivity of 100.0% (95% CI: 85.8–100.0) and a specificity of 56.9% (95% CI: 48.6–64.8). Positive and negative predictive values and likelihood ratios, with their corresponding 95% confidence intervals, are presented in Table 2.

### 3.3. Correlations Between BRIEF-MPI, G8, and Frailty-Related Measures

Significant correlations were observed between BRIEF-MPI, G8, and frailty-related measures (Table 3). Higher BRIEF-MPI total scores were moderately associated with greater frailty severity according to the Clinical Frailty Scale (CFS) (r_s_ = 0.545, *p* < 0.001), as well as poorer functional status measured by Katz ADL (r_s_ = −0.609, *p* < 0.001) and Lawton–Brody IADL (r_s_ = −0.533, *p* < 0.001).

Similarly, the G8 total score demonstrated moderate correlations with frailty and functional measures, including CFS (r_s_ = −0.597, *p* < 0.001), Katz ADL (r_s_ = 0.456, *p* < 0.001), and Lawton–Brody IADL (r_s_ = 0.578, *p* < 0.001). Older age showed weak but significant correlations with both screening instruments and frailty severity.

### 3.4. BRIEF-MPI Scores Across Clinically Relevant Oncological Subgroups

In exploratory unadjusted analyses, BRIEF-MPI total scores differed significantly according to clinically relevant oncological characteristics (Table 4). Participants with distant organ metastasis had higher BRIEF-MPI scores than those without metastasis (*p* = 0.001), patients treated with palliative intent had higher scores than those receiving curative treatment (*p* < 0.001), and participants with ECOG performance status ≥ 2 had higher scores than those with ECOG 0–1 (*p* < 0.001). These comparisons were unadjusted and should not be interpreted as demonstrating independent associations.

## 4. Discussion

This study provides preliminary evidence supporting the convergent and discriminative validity of the Turkish version of the BRIEF-MPI as a rapid multidimensional screening tool in older adults with cancer. While both instruments demonstrated good discriminative ability for identifying CFS-defined frailty, the DeLong test showed no statistically significant difference in overall AUC between BRIEF-MPI and G8. Rather than indicating superior overall discriminative performance, our findings suggest distinct screening profiles: the G8 showed higher sensitivity, whereas the BRIEF-MPI provided substantially higher specificity.

The choice of a geriatric screening tool in daily oncology practice depends heavily on the clinic’s specific needs and resources, particularly the delicate balance between sensitivity and specificity. The G8 tool is widely recommended for its high sensitivity, which aims to capture nearly all vulnerable patients [4]. However, its correspondingly lower specificity may pose a theoretical risk of a higher false-positive rate, which could potentially increase the referral burden on limited geriatric services and raise concerns regarding treatment pathways and resource utilization [9]. Our findings confirm this critical trade-off. The lower sensitivity of the BRIEF-MPI suggests it may miss a small proportion of vulnerable patients compared with the G8. Nevertheless, its higher specificity might theoretically reduce false-positive classifications compared with the G8. In resource-constrained oncology settings with limited geriatric specialist availability, using a more specific screening tool such as the BRIEF-MPI may help optimize referral pathways. Nonetheless, whether this translates into meaningful clinical efficiency, reduced resource burden, or enhanced cost-effectiveness was not directly evaluated in the present study and remains a theoretical hypothesis requiring formal investigation in future health-services research [8].

Frailty in geriatric oncology is a multidimensional syndrome that extends well beyond mere functional decline [13]. In our cohort, BRIEF-MPI positive classification (classes 2–3) was strongly associated with CFS-defined frailty. While the CFS is a rapid, well-validated tool based on clinical judgment, it remains a predominantly unidimensional scale [10,11]. The BRIEF-MPI, on the other hand, preserves the multidimensional structure of the full MPI, providing immediate insights into specific domains of impairment such as cognition, polypharmacy, and nutrition without requiring extensive evaluation time [5]. It should also be noted that the BRIEF-MPI incorporates functional and mobility domains, which conceptually overlap with the CFS, Katz ADL, and Lawton–Brody IADL; thus, our findings partly reflect this shared functional construct rather than entirely independent domain validation. Nevertheless, rapidly identifying these specific domains of impairment may help the oncology team guide personalized preventive programs and implement targeted supportive interventions, such as early nutritional counseling or deprescribing potentially inappropriate medications [13,14,15]. Landmark randomized trials have demonstrated that such CGA-guided management significantly reduces severe treatment-related toxicity, decreases unplanned hospitalizations, and optimizes overall patient trajectories [2,6,16,17].

The present study adds to the growing international literature on the BRIEF-MPI outside its original development setting. In its initial Italian validation study, the BRIEF-MPI demonstrated high diagnostic accuracy (AUC = 0.92), with a sensitivity of 82% and specificity of 88% when compared directly with the full MPI [5]. Similarly, the recently developed self-administered version (SELFY-BRIEF-MPI) yielded an AUC of 0.90, with a sensitivity of 70% and a specificity of 91.6% in a Southern European cohort [18]. Our findings in a Turkish geriatric oncology cohort are broadly consistent with these foundational European studies, particularly regarding the instrument’s hallmark characteristics: an acceptable sensitivity (75.0%) and high specificity (91.5%). Furthermore, our observed discriminative accuracy for CFS-defined frailty (AUC = 0.883) compares favorably with prognostic accuracies previously reported for the BRIEF-MPI in acute emergency departments (AUC = 0.72) and COVID-19 wards (AUC = 0.75) [6,7]. To the best of our knowledge, this is the first cross-cultural adaptation and clinical evaluation of the BRIEF-MPI, specifically tailored to an oncology population outside its original cultural context, thereby providing preliminary evidence supporting the clinical applicability and validity of the BRIEF-MPI in Turkish older adults with cancer.

We also observed a significant association between higher BRIEF-MPI scores and advanced disease characteristics, including distant metastasis, palliative treatment intent, and poorer ECOG performance status. This finding suggests that multidimensional vulnerability may reflect not only age-related decline but also the overall burden of cancer and its treatment [19,20,21]. In contemporary oncology practice, older adults receive a wide range of systemic therapies, including chemotherapy, immunotherapy, targeted therapies, and combination regimens, each associated with distinct toxicity profiles. In patients with limited physiological reserve, treatment-related adverse events may interact with pre-existing geriatric vulnerabilities and influence clinical outcomes [22,23,24]. Therefore, multidimensional screening tools such as the BRIEF-MPI may help identify vulnerabilities across functional and oncological subgroups, supporting individualized care planning for older adults with cancer.

Our study has several limitations that must be acknowledged. First, the cross-sectional, single-center design may limit the generalizability of our results to broader or more diverse oncology populations. Second, the frailty classification in this study was based on the CFS rather than a full, multidisciplinary gold-standard CGA. Although the CFS is widely utilized in geriatric oncology, it cannot substitute for a comprehensive geriatric evaluation. Furthermore, the inherent domain overlap between the functional components of the BRIEF-MPI and the comparative tools (CFS, ADL, IADL) means that the observed diagnostic accuracy and correlation metrics partly reflect construct overlap. Third, as previously mentioned, the limited sample size prevented us from conducting robust subgroup analyses by modern systemic treatment. Fourth, although the BRIEF-MPI is designed as a rapid screening instrument, specific feasibility metrics such as precise administration time, completion rates, and formal user burden were not quantitatively evaluated in this cohort. Finally, we did not assess longitudinal oncological outcomes such as treatment-related toxicity, unplanned hospitalizations, early treatment discontinuation, or overall survival; therefore, the predictive validity of the Turkish BRIEF-MPI regarding these crucial long-term endpoints remains to be determined by future cohorts.

## 5. Conclusions

This study provides preliminary evidence supporting the clinical performance and convergent/discriminative validity of the Turkish version of the BRIEF-MPI in older adults with cancer. Although its overall discriminative ability for frailty was comparable to that of the G8, its higher specificity suggests potential utility in clinical settings where reducing false-positive referrals is prioritized. Future prospective and multicenter implementation studies are needed to evaluate its clinical utility, prognostic value, and actual impact on geriatric referral pathways in routine oncology practice.

## Figures and Tables

**Table 1:** Baseline characteristics of the study population (n = 177).

Variable	Total Cohort (n = 177)
**Age, years**	72.5 ± 5.8
**Women, n (%)**	100 (56.5)
**Marital status, n (%)**	
Single	4 (2.3)
Married	124 (70.1)
Widowed	38 (21.5)
Divorced	11 (6.2)
**Educational level, n (%)**	
Illiterate	11 (6.2)
Literate (no formal education)	12 (6.8)
5–8 years	88 (49.7)
High school	30 (16.9)
University	36 (20.3)
**Living arrangement, n (%)**	
Alone	25 (14.1)
With spouse/partner	119 (67.2)
Other	33 (18.6)
**Most common cancer types, n (%)**	
Breast cancer	52 (29.4)
Colorectal cancer	23 (13.0)
Prostate cancer	21 (11.9)
Bladder cancer	15 (8.5)
Ovarian cancer	11 (6.2)
Other cancers	55 (31.1)
**Distant metastasis present, n (%)**	71 (40.1)
**Treatment status, n (%)**	
No prior antineoplastic treatment	14 (7.9)
Receiving radiotherapy	2 (1.1)
Active systemic treatment	75 (42.4)
Follow-up/surveillance	86 (48.6)
**Treatment intent, n (%)**	
Curative	105 (59.3)
Palliative	72 (40.7)
**Time since diagnosis, n (%)**	
<3 months	23 (13.0)
3–12 months	39 (22.0)
>12 months	115 (65.0)
**Antineoplastic treatment within past 5 years, n (%)**	88 (49.7)
**ECOG Performance Status, n (%)**	
PS0	76 (42.9)
PS1	77 (43.5)
PS2	14 (7.9)
PS3	9 (5.1)
PS4	1 (0.6)
**Clinical Frailty Scale (1–9)**	3.2 ± 1.4
**Katz ADL (0–6)**	5.4 ± 1.2
**Lawton–Brody IADL (0–8)**	6.9 ± 2.0
**Number of medications**	4.0 ± 2.8
**G8 score (0–17)**	13.7 ± 2.5
**BRIEF-MPI total score (0–1)**	0.22 ± 0.15
**BRIEF-MPI risk category, n (%)**	
Low risk (0.00–0.33)	146 (82.5)
Moderate risk (0.34–0.66)	30 (16.9)
High risk (≥0.67)	1 (0.6)
**BRIEF-MPI positive (class 2–3), n (%)**	31 (17.5)
**G8 vulnerability (≤14), n (%)**	90 (50.8)

Data are presented as mean ± standard deviation or n (%). ADL: Activities of Daily Living; IADL: Instrumental Activities of Daily Living; ECOG: Eastern Cooperative Oncology Group; BRIEF-MPI: Brief Multidimensional Prognostic Index.

**Table 2:** Discriminative and classification performance of BRIEF-MPI and G8 for identifying CFS-defined frailty.

Measure	BRIEF-MPI	G8
Continuous-score AUC (95% CI)	0.883 (0.797–0.969)	0.900 (0.849–0.951)
Predefined positive classification	BRIEF-MPI positive	G8 vulnerability
	(classes 2–3)	(≤14 points)
Positive screening, n (%)	31 (17.5)	90 (50.8)
Sensitivity, % (95% CI)	75.0 (53.3–90.2)	100.0 (85.8–100.0)
Specificity, % (95% CI)	91.5 (85.9–95.4)	56.9 (48.6–64.8)
PPV, % (95% CI)	58.1 (39.1–75.5)	26.7 (17.9–37.0)
NPV, % (95% CI)	95.9 (91.3–98.5)	100.0 (95.8–100.0)
LR+ (95% CI)	8.83 (5.00–15.59)	2.32 (1.93–2.78)
LR− (95% CI)	0.27 (0.14–0.55)	0.00 (not estimable)
χ^2^	63.5	26.8
*p* value	<0.001	<0.001
Cramer’s V	0.599	0.389

CFS-defined frailty was defined as a Clinical Frailty Scale score ≥ 5. Classification performance measures, including sensitivity, specificity, positive predictive value (PPV), negative predictive value (NPV), and likelihood ratios, were calculated using the predefined clinical classifications (BRIEF-MPI classes 2–3 and G8 ≤ 14 points). AUCs were derived separately from ROC analyses using the continuous BRIEF-MPI and G8 total scores. For ROC analysis, the direction of the G8 score was reversed so that higher values indicated greater vulnerability. LR+: positive likelihood ratio; LR−: negative likelihood ratio; AUC: area under the curve.

**Table 3:** Correlations between BRIEF-MPI, G8, and frailty-related measures.

Variable	BRIEF-MPI Total (r_s_)	*p* Value	G8 Total (r_s_)	*p* Value
Clinical Frailty Scale (CFS)	0.545	<0.001	−0.597	<0.001
Katz ADL	−0.609	<0.001	0.456	<0.001
Lawton–Brody IADL	−0.533	<0.001	0.578	<0.001
Age	0.259	0.001	−0.326	<0.001

Spearman’s rank correlation coefficients (r_s_) are shown. Higher BRIEF-MPI scores indicate greater multidimensional vulnerability, whereas lower G8 scores indicate higher vulnerability.

**Table 4:** BRIEF-MPI scores across clinically relevant oncological subgroups.

Variable	Group	n	BRIEF-MPI Median (IQR)	*p* Value
Distant metastasis	No	106	0.19 (0.13–0.31)	0.001
	Yes	71	0.25 (0.13–0.38)	
Treatment intent	Curative	105	0.19 (0.13–0.31)	<0.001
	Palliative	72	0.25 (0.13–0.38)	
ECOG performance	0–1	153	0.13 (0.06–0.19)	<0.001
	≥2	24	0.25 (0.19–0.50)	

BRIEF-MPI scores are presented as median (interquartile range). Group comparisons were performed using the Mann–Whitney U test. Higher BRIEF-MPI scores indicate greater multidimensional vulnerability.

## Data Availability

The data presented in this study are available on request from the corresponding author. The data are not publicly available due to privacy and ethical restrictions involving patient information.

## Supplementary Materials

The following supporting information can be downloaded at: https://www.mdpi.com/article/10.3390/healthcare14172813/s1, Supplementary File S1: Item-level source mapping and cross-cultural adaptation of the Turkish BRIEF-MPI [25,26,27,28,29]; Supplementary File S2: The finalized Turkish BRIEF-MPI form.
