# Supplementary material for: Turkish Cross-Cultural Adaptation of the BRIEF-MPI in Older Adults with Cancer: Convergent and Discriminative Validity Compared with the G8 and Clinical Frailty Scale
Source: Healthcare (Basel). 2026 Sep 2;14(17):2813. doi: 10.3390/healthcare14172813 (PMC13566576; doi:10.3390/healthcare14172813)
Supplement: Supplementary file 1 [file healthcare-14-02813-s001.zip › Supplementary File S1.pdf]

Supplementary Table S1: Item-level source mapping and cross-cultural adaptation of the Turkish BRIEF-MPI

Domain	Original source instrument/basis	Original BRIEF-MPI item	Turkish adaptation	Basis for Turkish adaptation
**Basic Activities of Daily Living (ADL)**	Katz ADL	Is the patient able to eat completely independently?	Hasta tamamen bağımsız olarak yemek yiyebiliyor mu?	Validated Turkish Katz ADL; Arik et al., 2015
		Is the patient able to get dressed by him/herself completely independently?	Hasta tamamen bağımsız olarak giyinebiliyor mu?	
		Does the patient exercise complete self-control over urination and defecation?	Hasta idrar ve gaita kontrolünü tamamen sağlayabiliyor mu?	
**Instrumental Activities of Daily Living (IADL)**	Lawton–Brody IADL	Is he/she able to phone?	Hasta telefon kullanabiliyor mu?	Validated Turkish Lawton IADL; Guzel et al., 2024
		Is he/she responsible for taking medication in correct dosages at correct time?	Hasta ilaçlarını doğru dozda ve doğru zamanda almayı kendi başına yönetebiliyor mu?	
		Is he/she able to take care of all shopping needs independently?	Hasta tüm alışveriş ihtiyaçlarını bağımsız olarak karşılayabiliyor mu?	
**Mobility**	Barthel mobility scale*	Is he/she able to get in and out of bed/chair alone?	Hasta yataktan veya sandalyeden yardımsız kalkıp oturabiliyor mu?	Validated Turkish Modified Barthel Index; Küçükdeveci et al., 2000
		Is he/she able to walk at least ten feet without any assistance (with/without walking aids)?	Hasta en az 3 metreyi (yardımlı veya yardımsız) yürüyebiliyor mu?	
		Is he/she able to go up and down stairs without assistance?	Hasta merdivenleri yardımsız inip çıkabiliyor mu?	
**Cognitive status**	SPMSQ	What are the date, month, and year?	Hasta bugünün tarihini (gün/ay/yıl) doğru olarak söyleyebiliyor mu?	Validated Turkish SPMSQ; Dikmeer et al., 2025
		How old are you?	Hasta kendi yaşını doğru olarak söyleyebiliyor mu?	
		Can you count backward from 20 by 3's?	Hasta 20'den başlayarak 3'er 3'er geri sayabiliyor mu?	

**Nutrition**	Modified MNA-SF	BMI <21 or ≥30	Vücut kitle indeksi (VKİ) <21 veya ≥30	Validated Turkish MNA; Sarikaya et al., 2015
		Has food intake declined over the past 3 months due to loss of appetite, digestive problems, chewing or swallowing difficulties?	Son 3 ayda iştahsızlık, sindirim sorunları, çiğneme veya yutma güçlüğü nedeniyle besin alımında azalma oldu mu?	
		Weight loss >3 kg in the last 3 months	Son 3 ayda 3 kg'dan fazla kilo kaybı oldu mu?	
**Comorbidity**	CIRS-derived Selfy-MPI	Number of diseases requiring chronic therapies	Kronik tedavi gerektiren hastalık sayısı (0–13)	Adapted according to the original BRIEF-MPI/Selfy-MPI structure†
**Medications in chronic therapy**	Standard MPI	Number of drugs (active substances)	Kronik tedavide kullanılan ilaç/etken madde sayısı	Direct adaptation of the original BRIEF-MPI item†
**Co-habitation**	Standard MPI	With family / institution / alone	Aile ile / kurumda / yalnız	Direct adaptation of the original BRIEF-MPI categories†

Notes. ADL, Activities of Daily Living; IADL, Instrumental Activities of Daily Living; SPMSQ, Short Portable Mental Status Questionnaire; MNA-SF, Mini Nutritional Assessment–Short Form; CIRS, Cumulative Illness Rating Scale; MPI, Multidimensional Prognostic Index.

* In the original BRIEF-MPI development study, the mobility domain comprises three items based on the Barthel mobility scale (transfer, walking, and stair climbing), with the selection of mobility items informed by the approach used in the MPI-InChianti study.

† The original BRIEF-MPI retained the comorbidity, number-of-medications, and co-habitation domains without substantive modification; comorbidity was derived from the CIRS/Selfy-MPI, whereas medication number and co-habitation followed the standard MPI structure.
