# Supplementary material for: Turkish Cross-Cultural Adaptation of the BRIEF-MPI in Older Adults with Cancer: Convergent and Discriminative Validity Compared with the G8 and Clinical Frailty Scale
Source: Healthcare (Basel). 2026 Sep 2;14(17):2813. doi: 10.3390/healthcare14172813 (PMC13566576; doi:10.3390/healthcare14172813)
Supplement: Supplementary file 1 [file healthcare-14-02813-s001.zip › Supplementary File S2.pdf]

Hasta Adı- Soy Adı: Hasta no:

Brief-MPI (Multidimensional Prognostic Index)

Türkçe Versiyon – Hekim/Araştırmacı Tarafından Uygulanan Form

Uygulama Talimatı:
Bu form hekim veya araştırmacı tarafından, hasta değerlendirilerek doldurulmalıdır. Her madde için uygun seçeneği işaretleyiniz.

---

1. Temel Günlük Yaşam Aktiviteleri (ADL)

1. Hasta tamamen bağımsız olarak yemek yiyebiliyor mu?
   ☐ 0 = Hayır ☐ 1 = Evet
2. Hasta tamamen bağımsız olarak giyinebiliyor mu?
   ☐ 0 = Hayır ☐ 1 = Evet
3. Hasta idrar ve gaita kontrolünü tamamen sağlayabiliyor mu?
   ☐ 0 = Hayır ☐ 1 = Evet

---

2. Araçsal Günlük Yaşam Aktiviteleri (IADL)

4. Hasta telefon kullanabiliyor mu?
   ☐ 0 = Hayır ☐ 1 = Evet
5. Hasta ilaçlarını doğru dozda ve doğru zamanda almayı kendi başına yönetebiliyor mu?
   ☐ 0 = Hayır ☐ 1 = Evet
6. Hasta tüm alışveriş ihtiyaçlarını bağımsız olarak karşılayabiliyor mu?
   ☐ 0 = Hayır ☐ 1 = Evet

---

3. Mobilite

7. Hasta yataktan veya sandalyeden yardımsız kalkıp oturabiliyor mu?
   ☐ 0 = Hayır ☐ 1 = Evet
8. Hasta en az 3 metreyi (yardımlı veya yardımsız) yürüyebiliyor mu?
   ☐ 0 = Hayır ☐ 1 = Evet
9. Hasta merdivenleri yardımsız inip çıkabiliyor mu?
   ☐ 0 = Hayır ☐ 1 = Evet

---

4. Kognitif Durum (SPMSQ)

10. Hasta bugünün tarihini (gün / ay / yıl) doğru olarak söyleyebiliyor mu?
    ☐ 0 = Doğru ☐ 1 = Hatalı
11. Hasta kendi yaşını doğru olarak söyleyebiliyor mu?
    ☐ 0 = Hayır ☐ 1 = Evet
12. Hasta 20’den başlayarak 3’er 3’er geri sayabiliyor mu?
    ☐ 0 = Hayır ☐ 1 = Evet

---

5. Beslenme Durumu (Modifiye MNA)

13. Vücut kitle indeksi (VKİ) < 21 veya ≥ 30
    ☐ 0 = Hayır ☐ 1 = Evet

14. Son 3 ayda iştahsızlık, sindirim sorunları, çiğneme veya yutma güçlüğü nedeniyle besin alımında azalma oldu mu?
    ☐ 0 = Hayır ☐ 1 = Evet

15. Son 3 ayda 3 kg'dan fazla kilo kaybı oldu mu?
    ☐ 0 = Hayır ☐ 1 = Evet

---

6. Komorbidite (CIRS Selfy-MPI)

Kronik tedavi gerektiren hastalık sayısı:_____ **(0–13 arası)**
